# Adherence to the World Cancer Research Fund/American Institute for Cancer Research lifestyle recommendations in colorectal cancer survivors: results of the PROFILES registry

**DOI:** 10.1002/cam4.791

**Published:** 2016-07-14

**Authors:** Renate M. Winkels, Linde van Lee, Sandra Beijer, Martijn J. Bours, Fränzel J. B. van Duijnhoven, Anouk Geelen, Meeke Hoedjes, Floortje Mols, Jeanne de Vries, Matty P. Weijenberg, Ellen Kampman

**Affiliations:** ^1^ Division of Human NutritionWageningen UniversityWageningenThe Netherlands; ^2^ Netherlands Comprehensive Cancer Organisation (IKNL)UtrechtThe Netherlands; ^3^ Department of EpidemiologyGROW–School for Oncology and Developmental BiologyMaastricht UniversityMaastrichtThe Netherlands; ^4^ Department of Health Sciences and the EMGO+ Institute for Health and Care ResearchVU University AmsterdamAmsterdamThe Netherlands; ^5^ Center of Research on Psychology in Somatic diseasesDepartment of Medical and Clinical PsychologyTilburg UniversityTilburgThe Netherlands

**Keywords:** Colon and rectal cancer, lifestyle recommendations, survivorship

## Abstract

We examined adherence to the eight The World Cancer Research Foundation/American Institute for Cancer Research (WCRF/AICR) recommendations on diet, physical activity, and body weight among colorectal cancer survivors, and whether adherence was associated with intention to eat healthy and with the need for dietary advice. Adherence to these recommendations may putatively reduce the risk of recurrence and death. Studies on adherence to these recommendations in colorectal cancer (CRC) survivors are lacking. Adherence was assessed in a cross‐sectional study among 1196 CRC survivors and could range between 0 (no adherence) and 8 points (complete adherence). Participants completed questionnaires on dietary intake, physical activity, and body weight. Prevalence Ratios were calculated to assess whether adherence to recommendations were associated with dietary intentions and needs. Twelve percentage of the survivors adhered to 6 or more recommendations; 65% had a score between >4 and 6 points; 23% scored no more than 4 points. The recommendation for to be modest with consumption of meat showed lowest adherence: 8% adhered; whereas the recommendation not to use dietary supplements showed highest adherence (75%). 18% reported a need for dietary advice, but this was not associated with adherence to recommendations. Survivors with higher adherence reported less often that they had received dietary advice, were less likely to have the intention to eat healthier, but reported more often that they had changed their diet since diagnosis. There is ample room for improvement of lifestyle recommendations in virtually all CRC survivors. A minor part of CRC survivors expressed a need for dietary advice which was not associated with adherence to the recommendations.

## Introduction

Survival rates of colorectal cancer (CRC) patients have risen over the past decades and will continue to rise 1. CRC survivors have a high risk for cancer recurrence, and a higher risk than the general population for chronic health conditions such as obesity, diabetes and cardiovascular disease 2, 3, 4. Elevated risks may be associated with clinical or genetic factors, but also with lifestyle factors such as diet, physical activity, and being overweight or obese 3. Increased physical activity and a healthy diet after diagnosis have been associated with reduced risk of cancer recurrence and mortality 5 and improved health‐related quality of life 6.

The World Cancer Research Foundation/American Institute for Cancer Research (WCRF/AICR) advises cancer survivors to follow the lifestyle recommendations for cancer prevention 7; these recommendations focus on body fatness, physical activity, foods and drinks that promote weight gain, plant‐based foods, meat products, alcoholic drinks, preservation/processing/preparation of foods, and dietary supplement use. Studies among adult survivors of childhood acute lymphoblastic leukemia 8 and predominantly breast cancer survivors 9, 10 showed that adherence to the recommendations generally was low: 2.9 out of a maximum score of 7 8, and 4 out of a maximum score of 7 9, 10. A study among 255 early cancer survivors of predominantly breast cancer showed that only 11% adhered to all the five lifestyle recommendations that were assessed in that study (physical activity, smoking, alcohol, fruit, and vegetable intake) 11.

Despite the high number of CRC survivors, research on CRC survivorship and adherence to the WCRF/AICR recommendations in limited 12. Recently, researchers from the EPIC study used prediagnostic data to assess the association between adherence to WCRF/AICR recommendations and CRC‐specific and overall mortality 13; higher adherence was associated with lower CRC‐specific and overall mortality 13. Nevertheless, that study could only use prediagnostic data on adherence, obtained ~6.4 years before the incidence of cancer; the study showed that average adherence to recommendations was less than 3 (of 6) recommendations for male survivors, and <4 (of 7) recommendations for female survivors.

Intentions and needs of CRC survivors regarding a healthy lifestyle are important to consider for future lifestyle promotion programs. The concern for a healthy diet increased more in the period shortly after diagnosis in gastric and colon cancer patients than in breast cancer survivors, as studied in a survey of 380 patients 14. More than half of the gastric and colon cancer patients in that survey indicated a need for dietary advice 14. In a New Zealand survey among 40 CRC survivors, ~60% of the CRC survivors indicated that the received dietary advice was too limited to meet their needs, and most of the participants were interested in receiving additional dietary advice, especially overweight and obese participants 15. Whether the intention to eat healthy and the need for dietary advice are associated with adherence to lifestyle recommendations remains unanswered in CRC survivors.

This study had two aims. The first aim was to evaluate adherence to the WCRF/AICR lifestyle recommendations in CRC survivors. The second aim was to investigate whether the degree of adherence to WCRF/AICR recommendations was associated with intention to eat healthy and the need for dietary advice regarding a healthy diet.

## Methods

### Study design

**Figure 1:**
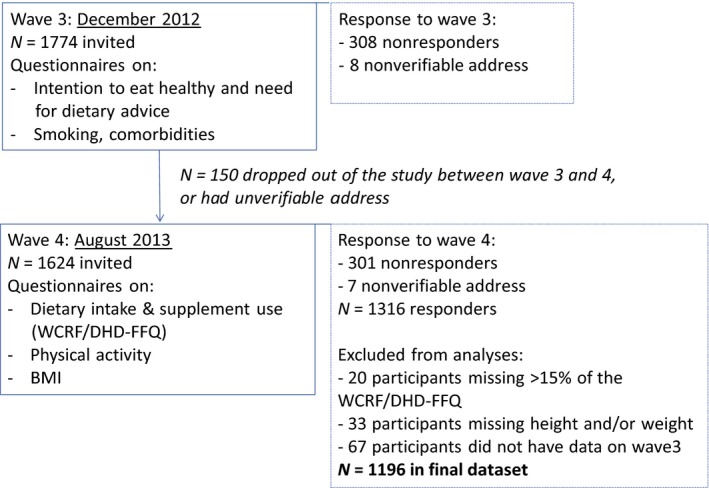
Flow diagram of study participants in a longitudinal study among colorectal cancer survivors, the PROFILES study. Figure S1 provides information on response to previous waves of this study, in this study information from waves 3 and 4 are used.

Data were collected within the PROFILES (Patient‐Reported Outcomes Following Initial Treatment and Long‐Term Evaluation of Survivorship) registry on physical and psychosocial impact of cancer and its treatment in cancer survivors and has been described in detail elsewhere 16. For this study, we used cross‐sectional data from CRC survivors participating in PROFILES 17 and the population‐based data of these survivors as collected within the Netherlands Cancer Registry. Participants were eligible when diagnosed with stage I–IV CRC between 2000 and 2009 and living in the south‐eastern part of the Netherlands. Exclusion criteria were as follows: having cognitive impairment or an unknown address. Participants were invited to respond to various questionnaires during four waves of data collection. This study used data from the third and fourth wave, see Figure**** 1. During the third wave in December 2012, data on intention to eat healthy, need for dietary advice, smoking, and comorbidities were collected. Data on dietary intake, supplement use, physical activity, and weight and height were collected in the fourth wave in August 2013. The study was approved by a local certified medical ethics committee; all participants signed informed consent.

A number of 1774 participants were invited for the third survey, see Figure**** 1. Between the third and the fourth survey, 150 participants either dropped out of the study, or were lost to follow‐up, rendering 1624 who were invited for wave four. Of these 1624, seven had an unverifiable address and 301 participants did not respond. Participants who completed <85% of the questions about dietary intake or physical activity (*n* = 20) and those with missing values for height and weight (*n* = 33) were excluded from the analysis. A number of 67 participants that responded to wave 4, had not responded to the third wave. Thus, the final dataset consisted of 1196 participants.

### Exposure assessment: adherence to the WCRF/AICR recommendations

We scored adherence to the 8 WCRF/AICR recommendations on: body fatness, physical activity, foods, and drinks that promote weight gain, plant‐based foods, meat products, alcoholic drinks, preservation/processing/preparation of foods, and dietary supplement use, see Table 1. Weight and height were self‐reported. Physical activity was assessed using the validated SQUASH‐questionnaire which contains questions about multiple activities referring to a normal week in the past months. Results were converted to time spent in light, moderate, and vigorous activities, which were then converted to activity scores 18. When this total activity score was 5 or more, representing the number of activities of at least 30 min per week, persons were categorized as adherent to the physical activity recommendation.

**Table 1:** The WCRF/AICR recommendations and the accompanying operationalization for each recommendation, followed by the percentage of survivors that adhered to this recommendation in a cohort of *n* = 1196 colorectal cancer survivors

WCRF/AICR score	Personal recommendations 7	Operationalization			Adherence[*n*, (%)]^a^
		0 points	0.5 points	1 points	
Body fatness	Ensure that body weight through childhood and adolescent growth projects toward the lower end of the normal BMI range at age 21	NA^b^	NA^b^	NA^b^	
	Maintain body weight within the normal range from age 21	<18.5 or >30 kg/m^2^	25 to <30 kg/m^2^	18.5 to <25 kg/m^2^	407 (34)
	Avoid weight gain and increases in waist circumference throughout adulthood	NA^b^	NA^b^	NA^b^	
Physical activity	Be moderately physically active, equivalent to brisk walking, for at least 30 min every day	<30 min	—	>30 min	886 (74)
	As fitness improves, aim for 60 min or more of moderate or for 30 min or more of vigorous, physical activity every day	NA^b^	NA^b^	NA^b^	
	Limit sedentary habits such as watching television	NA^b^	NA^b^	NA^b^	
Foods and drinks that promote weight gain	Consume energy‐dense foods (>225 to 275 kcal/100 g) sparingly	NA^b^	NA^b^	NA^b^	
	Avoid sugary drinks	Sugary drinks	—	No sugary drinks	697 (58)
	Consume fast food sparingly, if at all	NA^b^	NA^b^	NA^b^	
Plant foods	Eat at least five portions/servings (at least 400 g) of a variety of non‐starchy vegetables of fruits every day	Mean: F&V: <200 g/dayDietary fiber <8.5 g/day^b^^,^ c	Mean: F&V: 200–<400 g/dayDietary fiber 8.5–<17 g/day^b^^,^ c	Mean: F&V: ≥400 g/dayDietary fiber ≥ 17 g/day^b^^,^ c	113 (9)^d^
	Eat relatively unprocessed cereals and/or pulses with every meal	NA^b^	NA^b^	NA^b^	
	Limit refined starchy foods	NA^b^	NA^b^	NA^b^	
	People who consume starchy roots or tubers as staples also to ensure intake of sufficient nonstarchy vegetables, fruits, and pulses				
Meat products	People who eat red meat to consume less than 500 g/week, very little, if any, to be processed	Red/processed meat ≥500 g/week of which processed meat ≥50 g/day	Red/processed meat <500 g/week of which processed meat 3 to < 50 g/day	Red/processed meat <500 g/week of which processed meat <3 g/day	99 (8)
Alcoholic drinks	If alcoholic drinks are consumed, limit consumption to no more than two drinks a day for men, and one drink a day for women	♂:> 3 drinks♀:>2 drinks	♂:2 to ≤3 drinks♀:1 to ≤2 drinks	♂:≤2 drinks♀:≤1 drinks	881 (74)
Preservation, processing, preparation	Avoid salt‐preserved, salted or salty foods; preserve foods without using salt	NA^b^	NA^b^	NA^b^	
	Limit consumption of processed foods with added salt to ensure an intake of <6 g (2.4 g sodium) a day	Mean: >1.6 g/day^3^Always using discretionary sodium		Mean: ≤1.6 g /day^3^Never using discretionary sodium	149 (12)^d^
	Do not eat moldy cereals or pulses	NA^b^	NA^b^	NA^b^	
Dietary supplements	Dietary supplements are not recommended for cancer prevention	Use of supplements		No use of supplements	901 (75)

a Percentage of survivors completely meeting a single recommendation (1 point).

b Insufficient data available.

c Lowered by matching the percentage coverage of total energy intake as assessed by the WCRF/DHD‐FFQ (68%), see Methods for further explanation.

d For the recommendation of plant‐based foods, we averaged the adherence to the subrecommendations on fruits and vegetables and unprocessed cereals/grains. For the recommendation of preservation, we assessed intake of sodium from processed foods and discretionary salt; we assumed that 70% of salt intake comes from processed foods, whereas 30% comes from discretionary salt, see Methods for further explanation.

Dietary intake was assessed using an adapted version of the Dutch Healthy Diet – Food Frequency Questionnaire (DHD‐FFQ) 19, from now on referred to as the WCRF/DHD‐FFQ. The original DHD‐FFQ is a 34‐item screener to estimate adherence to the Dutch guidelines for a Healthy Diet during the previous month and is described in more detail elsewhere 19. The WCRF/AICR recommendations and the Dutch guidelines overlap in their recommendations on intakes of vegetables and fruit, dietary fiber, sodium, and alcoholic beverages; WCRF/AICR recommendations additionally include recommendations on meat products, and on foods and drinks that promote weight gain (Table 1). Therefore, we incorporated additional questions on sugary beverages, intake of meat, and processed meat. This adapted WCRF/DHD‐FFQ consisted of 40 items on intakes of bread, fruit, vegetable, potatoes, milk, cheese, meat products, fish, cookies, pastries, crisps, soup, fats, and oils, Asian take‐away food, pizza, sugary drinks, alcoholic beverages, and discretionary salt. Answer categories for frequency questions ranged from “never” to “every day.” Portion sizes were queried as standard, natural portions or as household measures such as glasses or bowls. Nutrient intakes were estimated by multiplying the portion sizes with frequencies and using the Dutch food composition table 20.

We operationalized adherence to the WCRF/AICR recommendations similar as to what Romaguera and colleagues did 21. If one of the recommendations was met, participants received 1 point for that recommendation. When a recommendation was not met; 0 or 0.5 points were allotted according to the available cut‐off values. For two recommendations – the recommendations of plant‐based foods and on preservation of foods, the score was allotted slightly different. For the recommendation on plant‐based foods, we averaged the adherence to the subrecommendations on fruits and vegetables and unprocessed cereals/grains. For example, if a participant partly met the recommendation on fruits and vegetables (0.5 points) and did not meet the recommendation for unprocessed cereals/grains (0 points), the total score for the recommendation on plant‐based foods for this participant was (0.5 + 0)/2 = 0.25. For the recommendation on preservation of foods, we focused on sodium intake. We assessed intake of sodium from processed foods and from discretionary salt; we assumed that 70% of salt intake comes from processed foods, whereas 30% comes from discretionary salt 22, 23. Thus, for example: a participant who was above the salt intake for processed foods (0 points), but who never added discretionary salt (1 point) to his foods, had an adherence score of (0 × 0.7 + 1 × 0.3) = 0.3.

The items of the WCRF/DHD‐FFQ covered ~68% of the absolute energy intake 24. Therefore, we lowered the cut‐off values to assess adherence to the recommendation for dietary fiber and for sodium intake proportionally. Thus, for dietary fiber intake, we assessed whether the dietary fiber intake was above the recommended level of 25 g/day × 68% = 17 g/day, and for sodium intake, we assessed whether the intake was below the level of 2.4 g of sodium/day × 68% = 1.6 g/day.

### Outcome assessment: intentions and dietary needs

Survivors were asked to respond to the following statements/questions: “I have the intention to adopt a healthier diet “ agree/disagree, “Did you change anything in your diet in the period since you were diagnosed with cancer” yes/no, “Did you received advice on dietary intake and/or use of dietary supplements after diagnosis” yes/no, and “If you did not receive dietary advice, did you feel a need for dietary advice” yes/no.

### Other covariates

Information on cancer diagnosis and cancer treatment history was provided by the Netherlands Cancer Registry (year of diagnosis, stage and localization of cancer, having a stoma). Socioeconomic status was based on the national economic value of residences and household income estimated from a fiscal database in 2000 and aggregated per postal code 25.

### Statistics

For each participant, adherence to the individual recommendations was calculated and summed, the maximum score was 8 (complete adherence). Total scores were categorized in four categories: a score of no more than 2 points, a score >2 and ≤4, a score >4 and ≤6 and a score >6.

Prevalence ratios (PR) were calculated to study the association between the prevalence of intention to adopt healthier diet and adherence to WCRF/AICR score; for the prevalence of dietary changes made since diagnosis and adherence; for the prevalence of having received dietary advice and adherence; and for the prevalence of a felt need for dietary advice and adherence. We calculated PR using Cox Regression instead of odds ratios using logistic regression. This was done because odds ratios overestimate the true association when the outcome of interest (in this case “intention to adopt healthier diet,” “dietary changes made since diagnosis,” “having received dietary advice,” or “need for dietary advice”) is not rare, whereas PR give a better estimation of the association. To calculate these ratios, we used Cox proportional hazard models with time fixed at 1 for each participant and with sandwich estimators of variance. In categorical models, we used the above mentioned categories for the WCRF/AICR‐score. In a continuous model, we included adherence to the WCRF/AICR score as a continuous variable to the model. The following variables were evaluated as possible confounding factors: gender, age, socioeconomic status (low, middle, high), tumor localization (colon or rectum), stage of the disease, stoma (yes/no), comorbidities (no, one or two or more), smoking status (current, former, never), and were included if they changed the HR by at least 10% using backward elimination; none of the possible confounding factors did. We explored whether associations differed between men and women by stratifying the analyses for gender, and by including an interaction term. All analyses were performed with SAS 9.3 (SAS Institute Inc., Cary, NC, USA) and STATA/SE 11.0 (Statacorp, Texas, USA) and *P* < 0.05 was considered statistically significant.

## Results

A number of 1624 colorectal cancer survivors was invited for wave 4; 1196 out of those 1624 (74%) participants were included in the final dataset, see Figure 1. Survivors who were not included in the final dataset were not meaningfully different (<5% different) from the participants in the final dataset with regard to age, gender, SES, time since CRC diagnosis, tumor stage, and tumor site (data not shown).

The mean score of adherence to the WCRF/AICR recommendations was 4.8 out of a maximum of 8. A number of 147 (12%) of the participants had an adherence score of more than 6; 774 participants had a score between 4 and 6 (65%); 267 participants (22%) scored between 2 and 4, and eight participants had a score of 2 or lower (1%). We merged the lowest two categories in the remaining analyses, as the number of participants in those categories was low, see Table 2.

**Table 2:** Characteristics of *n* = 1196 colorectal cancer survivors within categories of adherence to the WCRF/AICR recommendations score [*n* (%)]

	WCRF/AICR score		
	Cat 1^a^≤4 points	Cat 2>4 and ≤6 points	Cat 3>6 points
*n*	275	774	147
Gender			
Male	173 (63%)	447 (58%)	67 (46)
Female	102 (37%)	327 (42%)	80 (54)
Age			
<65 years	77 (28%)	197 (25%)	28 (19)
≥65 years	198 (72%)	577 (75%)	119 (81)
Socio‐economic status			
Low	57 (22%)	119 (16%)	29 (20)
Medium	106 (41%)	304 (41%)	52 (37)
High	98 (37%)	311 (42%)	61 (43)
Years since diagnosis			
<5 years	56 (20)	181 (23)	36 (24)
≥5 years	219 (80)	593 (77)	111 (76)
Tumor localization			
Colon	158 (57)	452 (58)	84 (57)
Rectum	117 (43)	322 (42)	63 (43)
Tumor stage			
Stage I	82 (30)	240 (31)	52 (35)
Stage II	85 (31)	265 (34)	55 (37)
Stage III	89 (32)	224 (29)	34 (23)
Stage IV	8 (3)	21 (3)	2 (1)
Stoma			
Yes	43 (16)	124 (16)	21 (14)
No	232 (84)	650 (84)	126 (86)
Comorbidities^a^			
0	55 (20)	176 (22)	37 (25)
1	72 (26)	201 (26)	45 (31)
≥2	148 (54)	397 (51)	65 (44)
Smoking^a^			
Current	29 (11)	61 (8)	17 (12)
Former	171 (63)	471 (62)	66 (47)
Never	70 (26)	223 (30)	59 (42)
Intention to eat healthier^a^			
Yes	106 (40)	233 (33)	39 (30)
No	157 (60)	497 (67)	91 (70)
Dietary changes made^a^			
Yes	62 (26)	189 (27)	51 (40)
No	181 (74)	500 (73)	77 (60)
Received dietary advice^a^			
Yes	58 (22)	122 (17)	18 (13)
No	207 (78)	616 (83)	124 (87)
Need for dietary advice^a^			
Yes	41 (20)	112 (17)	25 (20)
No	164 (80)	529 (83)	98 (80)

a Category 1 is a merged category of participants with an adherence of no more than 2 points (*n* = 8) and participants with an adherence a score >2 and ≤4 (*n* = 267).

Survivors with higher level of adherence were more likely to be women, older than 65 years, never‐smokers, diagnosed less recently, were diagnosed with a lower stage of cancer, and had slightly fewer comorbidities, see Table 2.

The degree of adherence to individual WCRF/AICR recommendations varied from 8 to 75%, see Table 1. None of the separate recommendations showed complete adherence by all participants. The ranking of recommendations from the lowest to the highest adherence was as follows: intake of red and processed meat (8% of the participants adhered to this recommendation), consumption of plant‐based foods (9%), salt intake (12%), body fatness (34%), foods and drinks that promote weight gain (58%), physical activity (73%), alcoholic drinks (74%), and no use of dietary supplements (75%).

Out of the total group of 1196 CRC survivors, 378 survivors (32%) agreed to the statement that they intended to adopt a healthier diet, 302 survivors (25%) stated that they changed something in their diet after diagnosis, 233 survivors (19%) of the survivors indicated they had received advice on dietary intake or use of dietary supplements after diagnosis, and out of the survivors that did not receive such advice 18% indicated that they felt the need for dietary advice (data not shown in Tables).

Adjusted PRs in Table 3 show that the prevalence of intention to adopt a healthier diet was lower among survivors with a higher adherence score to WCRF/AICR recommendations. Survivors in the categories of higher adherence reported more often that they had made changes to their diet after diagnosis. Survivors in the highest categories of adherence reported less often that they had received dietary advice after diagnosis. The need for dietary advice was not associated with adherence to WCRF/AICR recommendations. The results of analyses in which we included adherence to WCRF/AICR recommendations as a continuous score were consistent with the findings from the categorical analyses, see Table 3. PR appeared to be slightly more pronounced for men than for women, but p for interaction was not significant for any of the associations (data not shown).

**Table 3:** Prevalence ratios for intention to eat healthier, dietary changes made, having received dietary advice and need for dietary advice (95% CI) across categories of the WCRF/AICR score in a group of *n* = 1196 colorectal cancer survivors, with lowest adherence to the WCRF/AICR score as the reference category, and prevalence ratios with adherence to the WCRF/AICR score included as a continues score

	*n*	WCRF/AICR score in categories			Continuous WCRF/AICR score
		Cat 1≤4 points	Cat 2>4 and ≤6 points	Cat 3>6 points	
Prevalence of having the intention to eat healthier^a^	1105	1 (ref)	**0.82 (0.68, 0.98)**	0.74 (0.55, 1.01)	**0.88 (0.82, 0.95)**
Prevalence of dietary changes made since diagnosis^a^	1006	1 (ref)	1.09 (0.85, 1.39)	**1.54 (1.13, 2.09)**	**1.11 (1.01, 1.22)**
Prevalence of having received dietary advice^a^	1145	1 (ref)	0.76 (0.58, 1.00)	**0.56 (0.34, 0.92)**	**0.84 (0.75, 0.95)**
Prevalence of need for dietary advice^a^	969	1 (ref)	0.87 (0.63, 1.20)	0.93 (0.60, 1.44)	0.95 (0.83, 1.08)

a Adjusted for age and gender. Bold values are prevalence ratios that are statistically significant.

## Discussion

Our analyses within a large group of CRC survivors show that lifestyle and body weight of these survivors were not in agreement with the recommendations. Lowest adherence was shown for the recommendations on intake of red and processed meat and on consumption of plant‐based foods. Survivors with higher levels of adherence were less likely to have the intention to eat healthier, but reported more often that they had changed their diet since diagnosis. Moreover, they reported less often that they had received dietary advice.

The generally low overall adherence to the WCRF/AICR recommendations in our study was comparable to the findings in other types of cancer survivors 8, 9, 10, 11, 26. Scoring of adherence to the guidelines in those studies was not completely comparable to our study as in those studies 8, 9, 10, 11 adherence to guideline not to use supplements and to the guideline on foods and drinks that promote weight gain were not evaluated, whereas 2 points were allotted on the guideline for plant‐based foods. A study among cancer survivors of several types within the Multiethnic Cohort 26 reported a mean score of 3.7 out of the maximum of 6 points; dietary supplement use and intake of foods and drinks that promote weight gain were not evaluated. This mean score is very similar to the score found in our study: when we left out the subscores on supplement use and on intake of foods and drinks that promote weight gain, mean adherence in our data was 3.5 out of the maximum of 6 points (data not in tables). It is important to stress that we specifically studied a cohort of CRC survivors, whereas the earlier studies focused on survivors of several cancer types who may differ with respect to their ability to eat and to meet dietary guidelines 14, 27. Analyses in the EPIC study, showed that 51% of the CRC survivors adhered to <3 (out of 6) recommendations for men, and to <4 (out of 7) recommendations for women 13. Key difference with our study is that in the EPIC study prediagnostic adherence to WCR/AICR recommendations was assessed – on average 6.4 years before cancer incidence – while lifestyle of these persons may have changed. Furthermore, not all recommendations were evaluated in that study, while we were able to score adherence to all recommendations. Nevertheless, adherence to WCRF/AICR recommendations appears to be low among various types of cancer survivors.

The WCRF/AICR recommendations are not targeted toward specific types of cancer survivors, but are constructed to lower the overall risk of cancer 7. Yet, for prevention of colorectal cancer, the WCRF/AICR judged that the evidence was convincing that increased levels of physical activity, intake of foods containing dietary fiber/plant foods, low intake of red and processed meat, low intake of alcohol in men and low (abdominal) body fatness lowered the risk of CRC 28. Whether these factors also lower the risk of cancer recurrence for CRC survivors has not yet been fully elucidated, but may be plausible 3, 29. Our results show that for all those factors there is substantial room for improvement among CRC survivors.

CRC survivors with higher adherence to the recommendations less often reported the intention to adopt a healthier diet. This may indicate that survivors with a lower adherence know they could make healthier lifestyle choices. Interestingly, survivors in the highest category of adherence to recommendations more often reported they had changed their diet since diagnosis, but less often reported that they received dietary advice. This may suggest that survivors adopted a healthier lifestyle out of intrinsic motivation and not as a response to advice of a health professional. The overall need for dietary advice was expressed by 18% of the survivors, which is considerably lower than the 60% that is reported in an Italian study 14. This difference in need for dietary advice may be a result of cultural differences between Italy 14 versus the Netherlands, or in the way that the need for dietary advice was assessed.

Major strengths of our study are the large sample size and the high response rate, which can be mainly attributed to the well‐established PROFILES registry to facilitate the data collection 16. Nevertheless, survival bias may partly limit the generalizability of our findings, as a result of which the number of survivors with stage IV disease was low in our sample. Thus, one has to be cautious in extrapolating our findings to patients with stage IV disease. Moreover, data on lifestyle were self‐reported by survivors and may be prone to under or over reporting. If the respondents in our study indeed gave socially desirable answers, this would mean that the overall adherence to the lifestyle recommendations will even be lower than shown in our study. Another consideration is that the DHD‐ffq was validated as it was constructed 19, but the adapted WCRF/DHD‐FFQ was not validated as such. Nevertheless, we used the same methodology for the additional included items as during development of the original validated DHD‐ffq. As the adaptations to the original DHD‐ffq were minor, we expect that the validity of the present results would be similar 19. One last aspect to mention is that the information on intention to eat healthy and need for dietary advice were collected approximately half a year before we collected the data on adherence to WCRF/AICR recommendations. However, we assume that lifestyle and thus adherence to recommendations among long‐term survivors are relatively stable over this short time‐period and that this will not affect our results. A last consideration that needs to be taken into account is that we were able to include information on all the WCRF/AICR recommendations in our score, but that we did not have information to operationalize all the subrecommendations, as can be seen from Table 1. This should be considered when interpreting our findings and comparing those with findings from other studies. Other studies may have information on other (sub) recommendations, which may explain differences in findings between our study and others.

In conclusion, this study showed that there is ample room for improvement of lifestyle and body weight of CRC survivors on all aspects identified in the WCRF/AICR recommendations, and that virtually all CRC survivors have lifestyle factors that could be improved. Moreover, only a minor part of the CRC survivors expressed a need for dietary advice, which suggests that it is very important that lifestyle interventions should also target survivors who may not perceive they need to adopt a healthier lifestyle. Intervention studies are needed to study whether lifestyle advice would increase adherence to the WCRF/AICR recommendations and whether these changes will result in lower risks of cancer recurrence and chronic diseases.

## Conflicts of Interest

None declared.

## Supporting information


**Figure S1.** Flow diagram of study participants in a longitudinal study among colorectal cancer survivors, the PROFILES study. This figure shows the response rates of all previous waves of PROFILES; this manuscript includes information from waves 3 and 4.
