# Supplementary material for: Adherence to the World Cancer Research Fund/American Institute for Cancer Research lifestyle recommendations in colorectal cancer survivors: results of the PROFILES registry
Source: Cancer Med. 2016 Jul 14;5(9):2587–95. doi: 10.1002/cam4.791 (PMC5055169; doi:10.1002/cam4.791)

APPENDIX

Supplementary Figure 2: Flow diagram of study participants in a longitudinal study among colorectal cancer survivors, the PROFILES study. This figure shows the response rates of all previous waves of PROFILES; the current manuscript includes information from waves 3 and 4.

Supplementary figure for: ID CAM4-2016-02-0096 "Adherence to the World Cancer Research Fund / American Institute for Cancer Research lifestyle recommendations in colorectal cancer survivors: results of the PROFILES registry" Winkels et al

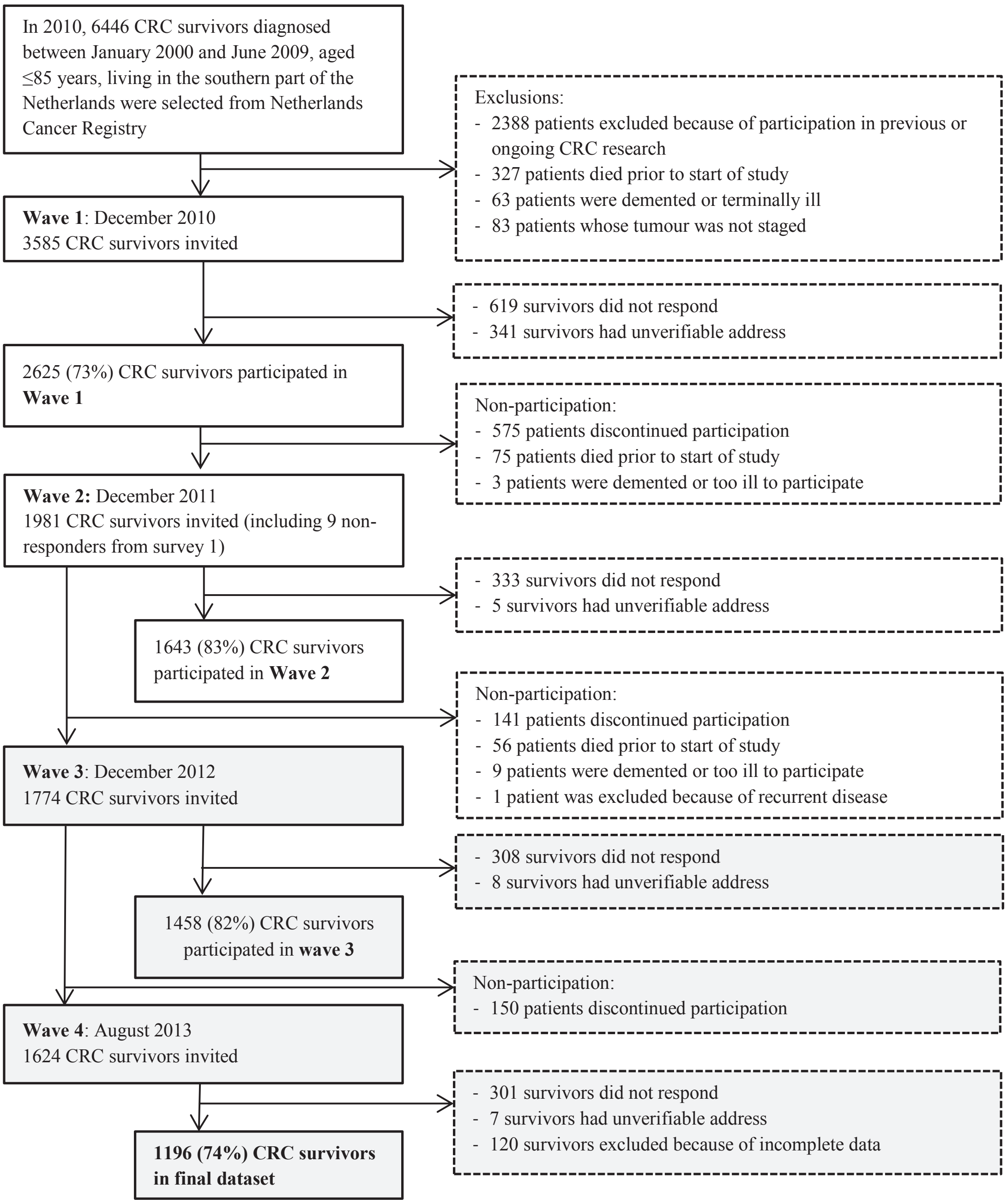

Supplement: Supplementary file 1 — Figure S1. Flow diagram of study participants in a longitudinal study among colorectal cancer survivors, the PROFILES study. This figure shows the response rates of all previous waves of PROFILES; this manuscript includes information from waves 3 and 4. [file CAM4-5-2587-s001.pdf]
